# Clinical course for patients with peritoneal carcinomatosis excluded from cytoreductive surgery and hyperthermic intraperitoneal chemotherapy

**DOI:** 10.1186/1477-7819-11-232

**Published:** 2013-09-16

**Authors:** Anne Peen Rodt, Rebekka Oxenvad Svarrer, Lene Hjerrild Iversen

**Affiliations:** 1 Department of Surgery P, Aarhus University Hospital, Tage Hansens Gade 2, Aarhus DK-8000, Denmark

**Keywords:** Colorectal cancer, Malignant mesothelioma, Goblet cell carcinoid, Pseudomyxoma peritonei, Treatment outcome, Postoperative complications

## Abstract

**Background:**

Cytoreductive surgery combined with 'Hyperthermic IntraPEritoneal Chemotherapy’ (HIPEC) represents the only potentially curative treatment available for carcinomatosis secondary to colorectal cancer (CRC), pseudomyxoma peritonei (PMP), malignant peritoneal mesothelioma (MM) and goblet cell carcinoma (GCC). Despite preoperative investigation some patients are excluded perioperatively because of unacceptably massive tumor extent. The data available on the clinical course of these patients are sparse. The aim of this study was to investigate mortality, morbidity and clinical course for patients who were excluded.

**Methods:**

This was a retrospective observational study based on records from 35 patients (21 men, 14 women) treated in a national center (Surgical Department P, Aarhus University Hospital) from June 2006 to August 2011 and excluded from the cytoreductive surgery perioperatively. The study population included patients aged 18 to 70 years with CRC (n = 19), PMP (n = 11), MM (n = 3) or GCC (n = 2). Vital status was obtained by 29 November 2012. Three patients were lost to follow-up.

**Results:**

The 30-day mortality rate was 0%. Postoperative complications within 30 days occurred in three patients (9.4%). In all, 19 patients (54%) had palliative surgery during exploratory laparotomy. In total, 28 patients (88%) received postoperative palliative chemotherapy. The median survival for CRC and PMP patients was 12.7 (95% CI 4.0 to 21.4) and 26.9 (95% CI 25.7 to 28.1) months, respectively.

**Conclusions:**

Exploratory laparotomy for intended curative treatment of peritoneal carcinomatosis did not imply major morbidity or mortality for patients excluded from treatment due to advanced stage of disease.

## Background

Peritoneal carcinomatosis (PC) represents the spread of malignancies to the parietal and visceral peritoneum. Cytoreductive surgery (CRS) and 'Hyperthermic IntraPEritoneal Chemotherapy’ (HIPEC) was introduced in the early 1990s as a potentially curative treatment in selected patients including PC secondary to colorectal cancer (CRC) and other malignancies such as pseudomyxoma peritonei (PMP) malignant peritoneal mesothelioma (MM) and goblet cell carcinoid (GCC) [1]. CRS aims to remove all visible tumor tissue (<2.5 mm). The HIPEC procedure is based on the principle that a high concentration of cytostatic drugs can eradicate the non-visible malignant cells in the upper layers of cells, while systemic absorption, and thereby toxicity, is limited. CRS and HIPEC may increase the 5-year survival up to 51% in selected patients with PC originating from CRC [2] and 87%-94% in patients with PMP [3,4].

The outcome of CRS and HIPEC depends on the tumor extent and the completeness of cytoreduction [5]. Selection of patients is challenging. Some patients deemed amenable to complete CRS and HIPEC may at time of surgery be excluded from CRS and HIPEC because of an unacceptably extensive tumor extent or unresectable disease, despite preoperative investigation with positron emission tomography-computed tomography (PET-CT). The literature on the clinical course for this group of patients is sparse. In this observational study, we present data on the clinical course and survival for patients found ineligible for CRS and HIPEC at time of surgery due to advanced stage PC.

## Methods

Patients referred to the Department of Surgery P, Aarhus University Hospital for suspected or diagnosed PC and potential need for CRS and HIPEC were registered prospectively from June 2006. The department is a national referral center for the CRS and HIPEC procedures [6].

Patients eligible for CRS and HIPEC were patients with PC originating from CRC, PMP, MM or GC. Exclusion criteria were: (1) physiological age above 70 to 75 years, (2) moderate and severe comorbidity as judged by an American Society of Anesthesiologists (ASA) score ≥III, (3) extraperitoneal disease, (4) invasive growth into the retroperitoneal space or the abdominal wall (except disease situated in previous incisions and port sites), (5) massive disease involvement of the small bowel or its mesentery, (6) more than one stenosis of the small bowel because of PC, (7) disease involvement of the hepatic pedicle or the pancreas, and (8) (for PC from CRC and appendiceal cancer only) PC extent in six or seven regions as estimated by Dutch 7 Region Count [7].

Preoperative assessment included CT/PET-CT of the lungs, abdomen and pelvis, and also colonoscopy if this had not been performed during the last year. From September 2010, diagnostic laparoscopy was also performed for the vast majority of patients. The overall completion rate of CRS and HIPEC increased from 56% to 70% after its introduction [8].

Treatment decisions were finalized in multidisciplinary team meetings. Final assessment of eligibility was performed at scheduled CRS and HIPEC during explorative laparotomy procedures, with staging of tumor extent according to the Dutch 7 Region Count Score [7] and evaluation of resectability.

From 1 June 2006 to 1 August 2011, 35 patients were excluded perioperatively from CRS and HIPEC.

In cases of unacceptably massive tumor extent or unresectable disease at time of scheduled CRS and HIPEC, standard palliative surgery was performed at the surgeon’s discretion, including resection or bypass of small bowel/colon/rectum when imminent obstruction was found omentectomy in severe intractable malignant ascites or where it was clinically indicated, and in patients with MM debulking was performed. When stabilized postoperatively, patients were transferred to their local hospitals. After discharge, these hospitals also received patients for readmissions, including secondary palliative surgery and palliative chemotherapy. Otherwise, these patients were treated by their general practitioner, public hospices, and by a public home nursing organization.

Patients were prospectively registered in connection with exploratory laparotomy including the reason(s) for perioperative exclusion from CRS and HIPEC. Data on postoperative complications and reoperations, readmissions and palliative surgery were collected retrospectively as of 15 December 2011 by review from E-journal, a Danish nationwide digital system of patient records. Postoperative infections were registered when verified, sepsis if symptoms and positive blood culture, wound infection if surgical debridement was needed, and pneumonia if verified by X-ray. Vital status was obtained as of 29 November 2012 and postoperative mortality (≤30 days) and overall survival were calculated. Total duration of hospitalization was registered for patients passed away as of 15 December 2011.

### Statistical analysis

Data were stratified according to primary diagnosis and given as median and range. In order to estimate time spent in hospital relative to residual lifetime after exclusion from CRS and HIPEC, readmission time was registered in percentage of survival for each patient succumbed at time for data collection (15 November 2011), and median and range of these data were calculated. Overall survival from time scheduled CRS and HIPEC was estimated using the Kaplan-Meier method. The Mann-Whitney-Wilcoxon test was used to estimate significance between two medians in the nonnormal small sample populations. P <0.05 was considered significant.

## Results

CRC and PMP constituted approximately one-half and one-third of patients, respectively (Table 1). The main reasons for exclusion comprised affection of more than five of seven peritoneal regions [7] for CRC patients and widespread PC of the small bowel or the mesentery for PMP patients (Table 2). About one-half of the patients presented ≥2 contraindications.

**Table 1:** Demographic data for patients excluded perioperatively from CRS and HIPEC (n = 35)

**Demographic**	**CRC**	**PMP**	**MM**	**GCC**	**Total**
Number of patients (%)	19 (54%)	11 (31%)	3 (9%)	2 (6%)	35 (100%)
Male/female	11/8	7/4	3/0	0/2	21/14
Age in years (median; range)	58 (22; 76)	57 (35; 76)	42 (38; 50)	61 (59; 63)	58 (22; 76)

*CRC* colorectal cancer, *CRS* cytoreductive surgery, *GCC* goblet cell carcinoid, *HIPEC* hyperthermic intraperitoneal chemotherapy, *MM* malignant peritoneal mesothelioma, *PMP* pseudomyxoma peritonei.

**Table 2:** Criteria for perioperative exclusion (n = 35)

**Criteria**	**CRC (n =19)**	**PMP (n =11)**	**MM (n =3)**	**GCC (n = 2)**
Involvement of >5 regions	9 (31%)	NA	NA	1 (50%)
Involvement of pancreas	2 (7%)	1 (7%)	1 (14%)	0 (0%)
Involvement of portahepatis	1 (3%)	5 (33%)	2 (29%)	1 (50%)
Stenosis of the ureter(s)	2 (7%)	0 (0.0%)	0 (0%)	0 (0%)
≥2 bowel stenoses	3 (10%)	0 (0.0%)	0 (0%)	0 (0%)
Widespread carcinomatosis in bowel/mesentery	5 (17%)	8 (53%)	3 (43%)	0 (0%)
Retroperitoneal involvement	3 (10%)	0 (0%)	0 (0%)	0 (0%)
Other^a^	4 (14%)	1 (7%)	1 (14%)	0 (0%)
Total number of reasons	29 (100%)	15 (100%)	7 (100%)	2 (100%)

^a^Other reasons included: liver metastases, para-aortal gland involvement, involvement of urinary bladder, severe adhesions and the stomach.

*CRC* colorectal cancer, *GCC* goblet cell carcinoid, *MM* malignant peritoneal mesothelioma, *PMP* pseudomyxoma peritonei.

Palliative surgery was performed in 19 patients (54%), and included omentectomy in 16 patients (40%), resections of colon or rectum in 7 patients and bypass procedures in 2 patients (Table 3).

**Table 3:** Palliative procedures performed during the exploratory laparotomy procedure (n = 35)

**Procedure**	**CRC (n = 19)**	**PMP (n = 11)**	**MM (n = 3)**	**GCC (n = 2)**
Omentectomy	6 (38%)	6 (43%)	3 (75%)	1 (20%)
Colon resection	3 (19%)	1 (7%)	0 (0%)	0 (0%)
Splenectomy	0 (0%)	1 (7%)	1 (25%)	0 (0%)
Resection of small bowel	1 (6%)	0 (0%)	0 (0%)	1 (20%)
Ileocecal resection	1 (6%)	3 (21%)	0 (0%)	1 (20%)
Bypass procedure	2 (13%)	0 (0%)	0 (0%)	0 (0%)
Gynecological procedures^a^	1 (6%)	0 (0%)	0 (0%)	1 (20%)
Rectal resection	0 (0%)	0 (0%)	0 (0%)	1 (20%)
Other procedures^b^	2 (13%)	3 (21%)	0 (0%)	0 (0%)
Total no. of procedures^c^	16 (100%)	14 (100%)	4 (100%)	5 (100%)
Total no. (%) of patients who underwent a palliative procedure	9 (47%)	6 (55%)	3 (100%)	1 (50%)

^a^Includes hysterectomy and salpingo-oophorectomy.

^b^Other procedures included: excision of the umbilicus, peritonectomy and operation for the correction of a diaphragmatic hernia.

^c^In all, 14 patients had ≥1 procedure performed.

*CRC* colorectal cancer, *GCC* goblet cell carcinoid, *MM* malignant peritoneal mesothelioma, *PMP* pseudomyxoma peritonei.

### Postoperative clinical course

The 30-day postoperative mortality after the exploratory laparotomy was 0%.

Postoperative complications within 30 days occurred in three patients (9.4%), who all received at least 2 palliative procedures (patient 1: Right hemicolectomy, omentectomy and resection of umbilicus. Patient 2: Omentectomy, gynocological procedures, small bowel resection, ileocaecal and rectum resection. Patient: 3 Omentectomy and ileocaecal resection). One patient had a rectal stump blowout after surgery. This was treated with drainage by Foley catheter. Another patient had an infected hematoma in the abdomen evacuated by surgery. A third patient received antibiotics for sepsis. A total of 11 patients had an anastomosis, 9 resections (including small intestines) and 2 bypass procedures; none of them experienced a leakage.

### Follow-up

Data on postoperative complications, readmissions and palliative surgery after discharge were missing for three patients, because postoperative data was not registered in E-journal, including two transferred to Greenland. Median follow-up regarding postoperative complications, readmission and palliative surgery for CRC patients were 9 months (range 1 to 36 months), PMP were 20 months (range 2 to 65 months), MM were 22 months (range 9 to 35 months) and GCC were 20 months (range 17 to 23 months). Vital status was available for all 35 patients. Follow-up regarding overall survival was longer with median values of 13 months (range 1 to 44 months) for CRC, 26 months (range 2 to 66 months) for PMP, 33 months (range 9 to 46 months) for MM, and 26 months (range 18 to 34 months) for GCC.

### Secondary hospitalization

Secondary operative intervention was needed in eight patients (25%) including three CRC patients, three PMP patients, and two GCC patients. Six of the eight patients had bowel obstruction, and decompression palliative procedures were performed in five. Two patients died within 30 days after the secondary operation, including one patient in whom surgery was deemed futile during operation. Three patients chose to have further surgery performed abroad (Germany, India and China, all with palliative outcomes), and were subsequently referred back to their local hospital in Denmark for postoperative care for median 24 days (range 14 to 42 days). Medical data from their hospitalization abroad was not available.

Apart from surgery and chemotherapy, treatment included a variety of palliative measures. For many patients, final terminal care was provided by public hospices.

A total of 13 (68%) CRC patients and 5 (45%) PMP patients were deceased at time for follow-up regarding the postoperative complications. These patients spent 7% (PMP) to 10% (CRC) of their remaining time in hospital.

### Postoperative chemotherapy

In total, 28 patients (88%) subsequently received palliative chemotherapy according to the Danish national guidelines [9]. In all, 14 CRC patients (82%) received chemotherapy (2 CRC patients were lost for follow-up). The median interval between the exploratory laparotomy and the initial chemotherapy was 29 days (20; 224). Nine PMP patients (90%) (one was lost to follow-up) received chemotherapy and the median interval until initiation of chemotherapy was 37 days (23; 714). All MM and GCC patients received chemotherapy. For MM the median interval was 43 days (29; 818) and GCC 29.5 (16; 43). Initiation of postoperative chemotherapy was delayed in patients who underwent palliative surgery during exploratory laparotomy as compared to those who had explorative laparotomy only. CRC patients who had explorative laparotomy had their first chemotherapy 28.5 (19;121) days after surgery, whereas CRC patients who received palliative procedures during explorative laparotomy had their first treatment 36.5 (20;224) days postoperatively. Similarly for PMP patients; 37 (25;714) days and 48 (23;64) days in those had not and had palliative procedures. The difference was not significant in either group (respectively P=0.66 and P=0.90).

### Survival

**Figure 1:**
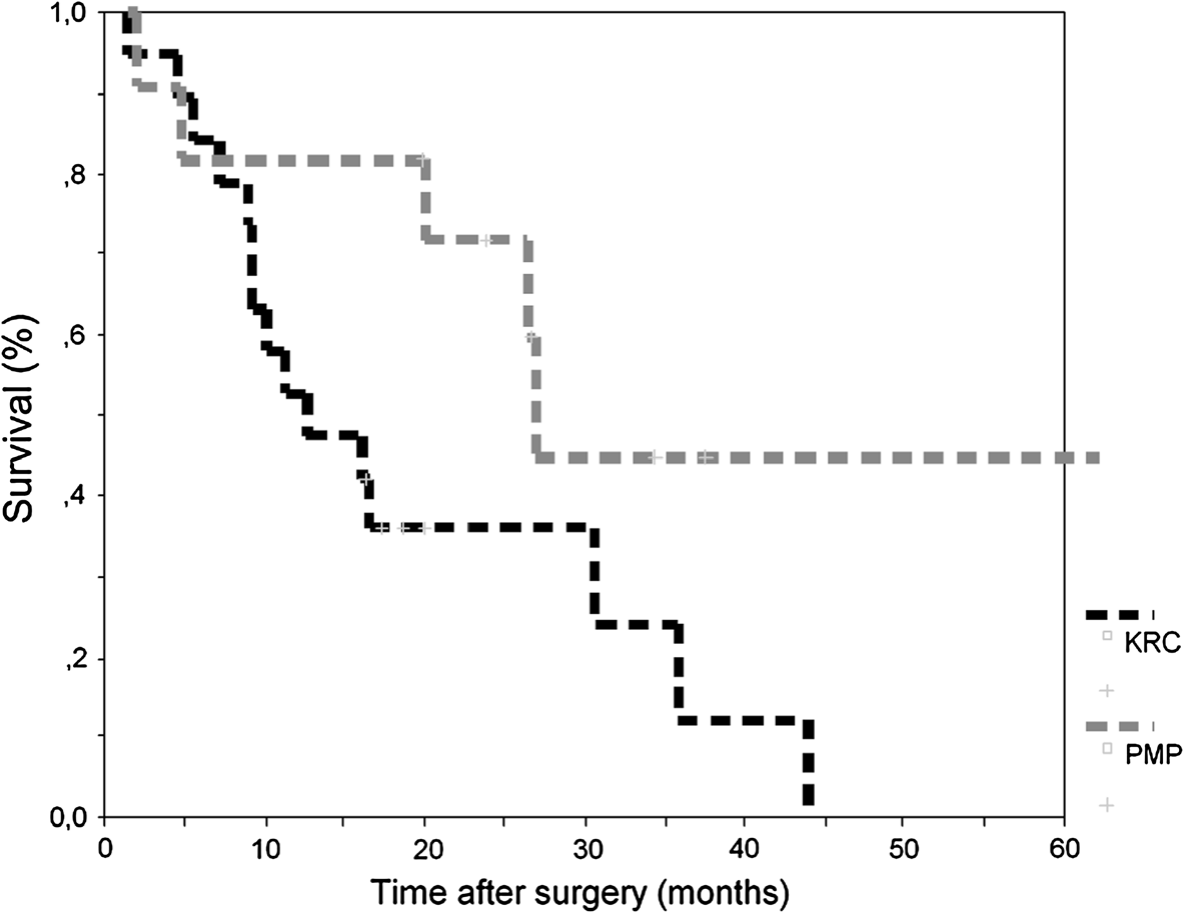
Kaplan-Meier curve for overall survival of patients with peritoneal carcinomatosis of colorectal origin and pseudomyxoma peritonei.

By 29 November 2012, 12 patients were still alive. Median survival was 12.7 months (95% CI 4.0 to 21.4) for CRC patients and 26.9 months (95% CI 25.7 to 28.1) for PMP patients, respectively (Figure 1). Median survival for patients with MM was not reached; two patients were still alive at 32.9 and 45.8 months, respectively, whereas the third patient died within 8.7 months. One GCC patient was alive 33.6 months after surgery and one passed away after 17.5 months.

## Discussion

This study showed a 30-day mortality rate of 0% and postoperative complications occurred in less than 10% of patients perioperatively excluded from CRS and HIPEC. In patients who only had exploratory laparotomy without palliative surgical intervention, no significant postoperative complications were observed. When implementing new treatment regimens, it is important to obtain information on the group of patients deemed potential candidates for the new treatment, but found ineligible following invasive investigation procedures, such as exploratory laparotomy. One might be concerned that the exploratory laparotomy inflicted unnecessary morbidity and mortality and affected overall survival and quality of life negatively compared with traditional treatments.

Patients not fulfilling the eligibility criteria for CRS and HIPEC represent a group with highly advanced PC. It might be expected that these patients displayed a rapid, fatal clinical course, but our results showed a median survival in the CRC group of 12.7 months. These findings are comparable to results found by Hompes *et al*. who found a median survival of 9.3 months for patients with PC who received systemic chemotherapy [10] as did 88% of patients in the current study. The difference in survival might be explained by the difference in the number of patients who received chemotherapy. However, it is more likely to assume the difference in survival is caused by selection bias since present patients were potential candidates for CRS and HIPEC, that is, without massive PC and fit for major surgery. A randomized prospective study by Bloehmendaal *et al*. [11] found an overall survival of 12.6 months for 50 patients with PC of colorectal origin randomized for standard treatment including simple chemotherapeutic regimens and not modern combination regimens. These patients were also highly selected and were therefore more comparable to the patients in this study. Present results do not indicate any reduction in survival of CRC patients despite an exploratory laparotomy, but our study is of limited size. Nevertheless, the median survival for CRC patients with PC as the only manifestation of disseminated illness is poor.

Data on survival for patients with PMP show marked variation. Youssef *et al*. reported that major debulking including hemicolectomy, omentectomy and splenectomy when needed, resulted in a median survival of 3.0 years (95% CI 2.2 to 3.8), however 29% of the patients received HIPEC as well and surgical palliative procedures were performed on a larger percentage of patients [3]. The 26.9 months survival in patients in the current study precluded from CRS and HIPEC is difficult to assess in this context. Furthermore, the classification of PMP and differentiation between high and low grade PMP remains a problem and a common understanding are desirable [12]. Firm data on the optimal treatment of PMP patients for whom complete cytoreduction is not possible are needed.

The patients received initial postoperative chemotherapy between 29 (CRC) and 43 (MM) days after exploratory laparotomy. No prospective randomized studies describing the optimal time interval from operation to postoperative chemotherapy exist. The current Danish standard treatment is based on the Multicenter International Study of Oxaliplatin/5-Fluorouracil/Leucovorin in the Adjuvant Treatment of Colon Cancer (MOSAIC) study [9] and recommends that patients should start treatment within 7 weeks. In present study, exploratory laparotomy did not delay the initiating chemotherapy as all patients fulfilled the MOSAIC requirements. However, it was noted that patients who underwent palliative surgery in general started chemotherapy eight (CRC) and 11 (PMP) days later then the patients who only had exploratory laparotomy. However this difference was not significant. One could suggest that the difference in duration between operation and chemotherapy across patient groups might be explained by the existence of a nationally defined cancer package for CRC patients [13].

It is encouraging that deceased patients spent only approximately 10% of their residual lifetime in hospital, independent of tumor origin. These positive figures should be interpreted in light of the well-developed primary public health care system comprising general practitioners, palliative teams as well as possibilities for final terminal care in hospices. The patient population studied was heterogeneous due to different underlying diseases. Therefore, we analyzed data categorized by primary disease making the groups even smaller. This is especially true for patients with MM and GCK (three and two patients), both of which are rare diseases. Therefore, we focussed mainly on the outcome of CRC and PMP patients.

The collection of data suffered the natural limitations of a retrospective study. E-journal is a nationwide digital system of patient records. It was introduced in 2005, but the frequency of reporting still varies between regions and departments. However, when departments report to E-journal, then all data from the patient records are reported. Postoperative complications may be underreported in the patient records; however, the registration of overall survival was not influenced. Patients in the present study include a selected group of PC patients as it was expected that curative CRS and HIPEC could be performed. Patients with poor physical condition and massive PC extent had already been excluded. This could affect the results towards a better outcome.

## Conclusions

This study showed that exploratory laparotomy prior to CRS and HIPEC is not associated with considerable morbidity or mortality for patients being perioperatively excluded from CRS and HIPEC. Nor do following palliative surgical procedures appear to be related to sustained postoperative morbidity and these procedures do not significantly delay potential postoperative chemotherapy. Seemingly, overall survival is not reduced despite exploratory laparotomy.

## Consent

According to Danish law this kind of project is classified as quality assurance project and written informed consent is not required for this kind of project.

## Abbreviations

CRC: colorectal cancer; CRS: cytoreductive surgery; CT: computed tomography; GCC: goblet cell carcinoid; HIPEC: hyperthermic intraperitoneal chemotherapy; MM: malignant peritoneal mesothelioma; PC: peritoneal carcinomatosis; PET-CT: positron emission tomography-computed tomography; PMP: pseudomyxoma peritonei.

## Competing interests

The authors declare that they have no competing interests.

## Authors’ contributions

Study concepts: APR, ROS, LHI. Study design: APR, ROS, LHI. Data acquisition: APR, ROS. Data analysis and interpretation: APR, ROS, LHI. Statistical analysis: APR, ROS, LHI. Manuscript preparation: APR, ROS. Manuscript editing: APR, ROS, LHI. All authors read and approved the final manuscript.
